# Biological Safety and Efficacy of the Novel Preservation Solution Ecosol in a Rat Liver Transplantation Model

**DOI:** 10.3390/ijms27010144

**Published:** 2025-12-23

**Authors:** Kerim Yildirim, Hirokazu Tanaka, Benedict M. Doorschodt, Kenji Fukushima, Shintaro Yagi, Felix Oldhafer, Oliver Beetz, Christian Bleilevens, Zoltan Czigany, Rene H. Tolba

**Affiliations:** 1 Institute for Laboratory Animal Science and Experimental Surgery, University Hospital RWTH Aachen, 52074 Aachen, Germany; kerim.yildirm@hotmail.de (K.Y.); hi-tank@kuhp.kyoto-u.ac.jp (H.T.); bd@vivalyx.com (B.M.D.); k.fukushima825@gmail.com (K.F.); 2 Department of Hepatobiliary, Pancreas and Transplant Surgery, Kyoto University Graduate School of Medicine, Kyoto 606-8303, Japan; 3 Division of Hepato-Biliary-Pancreatic Surgery, Department of Surgery, Kobe University Graduate School of Medicine, Kobe 650-0017, Japan; 4 Department of Hepato-Biliary-Pancreatic Surgery and Transplantation/Pediatric Surgery, Kanazawa University Graduate School of Medical Sciences, Kanazawa 920-8640, Japan; shintaro@kuhp.kyoto-u.ac.jp; 5 Department of General, Visceral, Pediatric and Transplant Surgery, Faculty of Medicine, University Hospital RWTH Aachen, 52074 Aachen, Germany; foldhafer@ukaachen.de (F.O.); obeetz@ukaachen.de (O.B.); 6 Department of Anesthesiology, Faculty of Medicine, University Hospital RWTH Aachen, 52074 Aachen, Germany; cbleilevens@ukaachen.de; 7 Department of Surgery, University of Heidelberg, 69120 Heidelberg, Germany; zoltan.cziganymd@gmail.com

**Keywords:** static cold storage, liver transplantation, university of wisconsin, preservation solution, organ preservation, rat, allogenic, syngeneic

## Abstract

Static cold storage remains the most widely used method for organ preservation in transplantation. Over time, preservation solutions have undergone continuous optimization. Ecosol is a novel extracellular-type, colloid-based preservation solution. In this study, we evaluated the safety and efficacy of Ecosol in comparison to the gold standard University of Wisconsin (UW) solution using both allogeneic and syngeneic rat orthotopic liver transplantation models. Liver function parameters were assessed and compared to baseline values of the respective rat strains. In the syngeneic setting, alanine transaminase (ALT) levels were significantly higher in the UW group than in the Ecosol group on day 1 post-transplantation (*p* < 0.05). Lactate dehydrogenase (LDH) levels were significantly elevated in the UW group compared to Ecosol in both allogeneic and syngeneic models on day 1 (*p* < 0.001). Gamma-glutamyl transferase (GGT) and total bilirubin were also significantly higher in the UW syngeneic group on day 7 (*p* < 0.05). In the allogeneic setting, aspartate aminotransferase (AST) and ALT levels were significantly elevated in both the UW (*p* < 0.0001) and Ecosol (*p* < 0.0001 and *p* < 0.001, respectively) groups on day 1 compared to the baseline values of Brown Norway (BN) rats. On day 7, these elevations persisted in the UW group, whereas no significant differences were observed in the Ecosol group compared to the baseline BN values (UW vs. Ecosol: *p* < 0.0001). In syngeneic transplants, AST and ALT levels were significantly elevated in both groups on day 1 compared to the baseline values of Lewis rats (*p* < 0.0001). By day 7, AST levels remained significantly elevated in the UW group, while Ecosol showed no significant difference from baseline (*p* < 0.0001). Organ viability, assessed via non-invasive imaging after 8 h of cold storage, was improved with Ecosol. Overall, Ecosol demonstrated biological safety and non-inferiority to the UW solution for liver preservation in a rat orthotopic liver transplantation model.

## 1. Introduction

Over the past two decades, liver transplantation has emerged as an essential therapeutic intervention for patients with acute liver failure and end-stage liver disease. Current five-year survival rates exceed 70%, highlighting the critical role of transplantation in clinical practice, particularly considering the lack of alternative treatments that provide comparable outcomes [1].

In 2024, a total of 1529 patients were listed as potential recipients for liver transplantation in Germany. Of these, 890 liver transplants were performed. Unfortunately, 288 patients on the waiting list died, and an additional 213 were removed due to various reasons, such as disease progression [2].

The persistent shortage of donor organs remains a major challenge in transplantation medicine, irrespective of the organ type. Several strategies have been developed to expand the donor pool. One such approach involves the use of extended criteria donors (ECDs), particularly in liver transplantation, where the acceptance criteria for donor organs are broadened [3].

Additional strategies to address organ shortage include the utilization of donation after brain death (DBD) and donation after cardiac death (DCD). DBD is standard and the only legally allowed method for post-mortem organ transplantation in Germany. In countries where brain death criteria are not universally accepted due to cultural or religious considerations, DBD remains controversial. Alternative approaches such as living donor liver transplantation (LDLT) and split-liver transplantation have been additionally introduced. Both methods allow for the transplantation of liver segments, enabling two recipients to benefit from a single donor organ [4].

Irrespective of the donor source, early graft dysfunction continues to represent a major challenge in liver transplantation. The procedure inherently involves the interruption of hepatic perfusion, which precipitates ischemic injury. This ischemia can induce irreversible cellular damage and may ultimately lead to primary graft dysfunction or nonfunction following transplantation. [5]. Two types of ischemic injury are distinguished: warm ischemic injury, which occurs at 37 °C body temperature from the time of interrupting donor liver perfusion after cardiac standstill and during explantation, and cold ischemic injury, which compromises the liver during cold storage, usually at 0-4 °C. Another paradoxical consequence of organ transplantation is reperfusion injury, which happens as soon as transplantation is performed, and the donor organ is re-perfused with 37 °C warm blood. Many pathophysiological mechanisms seem to contribute to this phenomenon, including lack of energy substrates, vascular leakage, inflammation, and transcriptional reprogramming [6]. Given the importance of ischemia–reperfusion injury and its contribution to possible early graft dysfunction, the significance of organ preservation has been emphasized. Organ preservation is thus considered a target for improvement to minimize tissue damage and maximize donor organ viability [7]. Many concepts were suggested to improve donor liver preservation, with one of them being the use of ex vivo machine perfusion, first applied by Carrel and Lindbergh in 1935 [8]. The intention, however, to mimic physiological conditions under normothermia or decrease the risk for early graft dysfunction by performing machine perfusion under hypothermic conditions faced limitations such as high costs, technical complexity, and the limited availability of blood products for perfusion [9]. Today, static cold storage (SCS) is still the standard method in liver preservation for transplantation. Several preservation solutions have been developed to minimize the negative impacts of cold and warm ischemic injury during a transplant procedure [10].

Currently, University of Wisconsin (UW) and Histidine-Tryptophan-Ketoglutarate (HTK) are the most widely distributed solutions used for organ preservation, whilst yielding similar results in several studies [11]. Ischemia- and reperfusion-associated injury to liver grafts remain the main reason for organ dysfunction during the transplantation procedure. This is combined with the inflammatory activation of the transplant on cellular as well as molecular levels [12]. Moreover, since the introduction of the currently established preservation solutions in the 1980s, no substantial advances in organ preservation have been achieved [13].

Ecosol, a novel preservation solution, has been developed in our laboratory and showed promising results in a study by Kalenski et al. using the isolated perfused porcine kidney model compared to HTK [14]. Ecosol is a colloid-based, extracellular-type solution made for use during SCS, hypothermic machine perfusion, and venous oxygen persufflation (Table 1). It contains amino acids, vitamins, buffer systems, and potent antioxidants to scavenge free oxygen radicals. Additional components are polyethylene-glycol (PEG), which is known to provide an immune-camouflage and reduce immunological organ rejection in recipients [15]. The aim of the current study was to evaluate Ecosol solution in a rat liver transplant model, comparing it to the widely used UW solution, as well as to show its biological safety and efficacy in vivo.

## 2. Results

Mean graft liver weight, mean recipient body weight, and mean postoperative body weight were 9.8 g ±0.9, 207.9 g ±18.5, and 204.8 g ±20.2, respectively. Mean cold ischemic time, mean warm ischemic time, and mean operation time were 510 min ±42, 19 min ±3, and 113 min ±14, respectively.

### 2.1. LDH and Liver Function Panel on Day 1 and Day 7 Post-Transplantation

**Figure 1:**
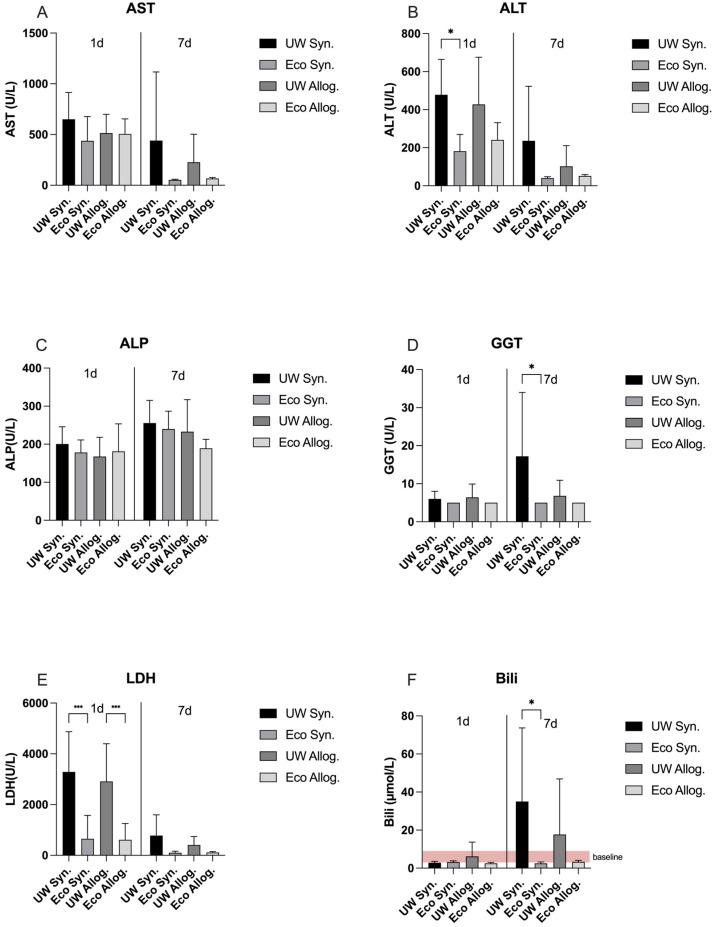
No differences in AST (**A**) or ALP levels (**C**) between groups and postoperative days. Significantly lower ALT values on day 1 (**B**), GGT concentration on day 7 (**D**), and total bilirubin on day 7 (**F**) if treated with Ecosol compared to UW in the syngeneic group. On day 1, both allogenic and syngeneic groups showed significantly lower LDH values (**E**) in the Ecosol group vs. UW. * *p* < 0.05; *** *p* < 0.001. Two-way ANOVA.

On day 1, ALT values were significantly lower in Ecosol compared to UW and the syngeneic groups (*p* < 0.05). Additionally, LDH results were significantly lower for Ecosol compared to UW in both syngeneic and allogenic groups (*p* < 0.001). The remaining liver panel did not show any statistically significant difference among both groups on day 1. On day 7 post-transplantation, both GGT and Bilirubin values in Ecosol treated liver displayed statistically significant lower results compared to UW in syngeneic groups (*p* < 0.05). The remaining liver panel did not show any statistically significant difference for both groups on day 7 (Figure 1).

### 2.2. Liver Panel Compared to Baseline in Allogeneic Groups

**Figure 2:**
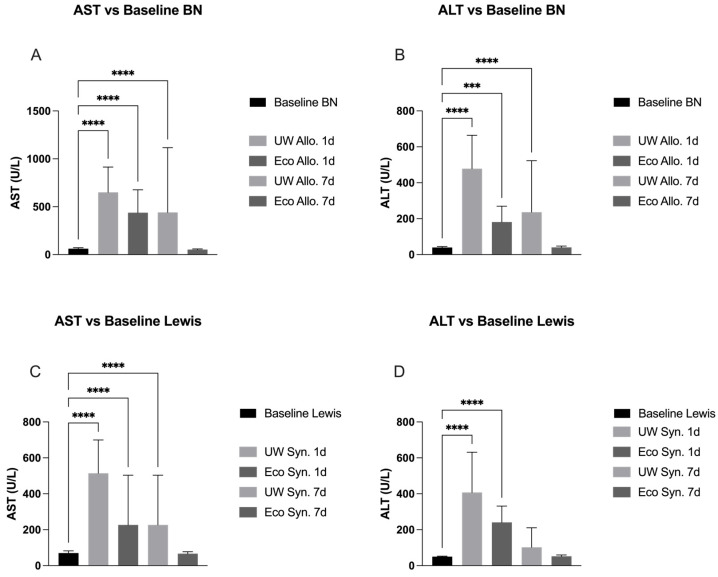
Significantly higher AST (**A**) and ALT values (**B**) on day 1, in both groups (Ecosol and UW) compared to baseline; both markers remained significantly higher in the UW group until day 7 and decreased back to baseline in the Ecosol group. For Lewis rats, on day 1, AST (**C**) and ALT values (**D**) were significantly elevated in both groups (Ecosol and UW) compared to baseline, and AST values remained significantly increased in the UW group until day 7. *** *p* < 0.001, **** *p* < 0.0001; Baseline values for Lewis rats: ©2019 Janvier Labs; baseline values for Brown Norway rats. One-way ANOVA.

AST on day 1 post-transplantation was significantly higher among Ecosol and UW compared to the baseline AST values of BN rats (*p* < 0.0001). On day 7 post-transplantation, the same statistical significance remained for UW while no difference was observed for Ecosol (*p* < 0.0001). ALT on day 1 post-transplantation was significantly higher for both Ecosol and UW compared to baseline (*p* < 0.001 and 0.0001, respectively). On day 7, UW remained significantly higher compared to baseline while Ecosol did not show any significant difference (*p* < 0.0001) (Figure 2).

### 2.3. Liver Panel Compared to Baseline in Syngeneic Groups

AST values among both Ecosol and UW were significantly higher on day 1 post-transplantation compared to baseline in Lewis rats (*p* < 0.0001). The same significance remained on day 7 after transplantation for the UW group, while no difference was observed for Ecosol (*p* < 0.0001). ALT values for both Ecosol and UW were significantly higher on day 1 post-transplantation compared to baseline (*p* < 0.0001). No significant difference was observed for both Ecosol and UW on day 7 post-transplantation (Figure 2).

### 2.4. TUNEL Assay

**Figure 3:**
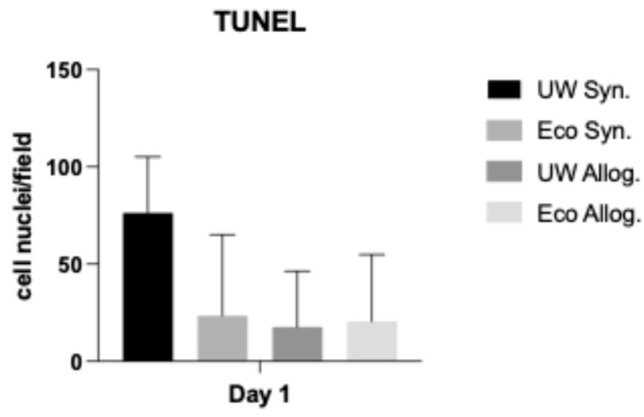
TUNEL Assay at day 1 (24 h) after transplantation. Demonstrated are positive cell nuclei per high power field on day 1 after transplantation. There was no statistically significant difference among both allogenic and syngeneic groups. One-way ANOVA.

The TUNEL (terminal deoxynucleotidyl transferase biotin-dUTP nick end labeling) Assay did not show any statistically significant differences for both Ecosol and UW in allogenic and syngeneic settings (Figure 3).

### 2.5. IVIS Imaging

**Figure 4:**
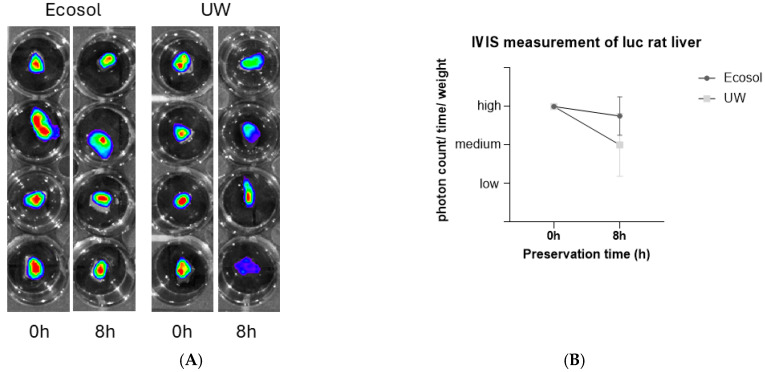
**(A**) Illustration of IVIS imaging from liver samples of luciferase expressing Lewis rats when using Ecosol and UW for cold storage for 8 h. Red color indicates higher intensity, followed by yellow color, green color and blue of luciferase expression. Ecosol treated livers express higher intensity of luciferase compared to UW. (**B**) IVIS measurement of Luciferase expressing Lewis transgenic rats demonstrating photon count/time/weight after 8 h of cold storage in Ecosol and UW. Higher photon count/time/weight was observed among Ecosol samples.

We utilized livers from Luciferase expressing transgenic Lewis rats to analyze the organ quality after preservation by using the IVIS imaging system. Liver samples of 2 mm diameter were obtained and preserved in Ecosol or UW solution for 8 h. Ecosol treated livers displayed stronger expression of luciferase after 8 h of cold storage compared to UW. IVIS measurements resulted in a higher photon count/time/weight for Ecosol compared to UW after 8 h of cold storage (Figure 4).

## 3. Discussion

Liver transplantation remains the only curative option for end-stage liver disease patients, as there are no comparable, extracorporeal replacement techniques available such as hemodialysis in chronic kidney disease. Cold ischemia and reperfusion injury during transplantation are major risks for donor organ viability. The length of the cold ischemic time can vary and is known to be negatively associated with graft dys- or nonfunction after transplantation [19]. Although there are alternate ways to static cold storage, such as hypothermic or normothermic machine perfusion, which showed superiority to SCS in several studies [20], SCS is still dominant in the clinical setting due to its cost-effectiveness [21]. Considering these findings, the preservation solution employed during static cold storage (SCS) has become a critical focus for optimizing graft quality in organ transplantation. UW solution is considered the clinical standard in liver graft preservation during SCS [22], although HTK is similarly effective and thus also widely used [11]. Newer preservation solutions are needed to improve graft quality and reduce the negative impacts of cold ischemic injury during SCS.

Our study aimed to evaluate the biological safety and efficacy of the novel preservation solution Ecosol compared to the clinical-gold standard UW solution in a rat liver transplantation model. Donor livers were perfused with the respective solutions and cold stored at 4 °C for 8 h in syngeneic or allogenic transplantation models afterwards. A key finding of this study was that Ecosol was non-inferior to UW. In several parameters, Ecosol performed better than the UW solution after orthotopic liver transplantation in rats.

Liver grafts in both the syngeneic and allogenic groups showed lower LDH values with the use of Ecosol compared to UW on day 1 and day 7 post-transplantation. Although LDH is an unspecific marker for cellular damage and injury by means of hypoxia, we must address these results obtained in our study. These findings suggest that Ecosol treated livers were better protected during SCS and IRI compared to UW.

Even though a significant difference was not observed for the entire liver panel on day 1 as well as day 7 post-transplantation, a clear tendency in all obtained parameters can be seen, which show a trend in four obtained values that represent hepatocellular and biliary tract injury in particular (ALT, AST, ALP, and GGT), favoring Ecosol over UW in both the syngeneic and allogenic groups. The observed variability, as reflected by the standard deviations, may account for the lack of statistical significance in some of the parameters discussed.

Our results portray the impact of preservation solutions on early graft function despite immunosuppressant usage among the allogenic groups, as both syngeneic and allogenic models display a similar tendency towards Ecosol. The tendency favoring Ecosol treated liver grafts among both groups represented by the liver enzyme panel remained after 7 days of transplantation. Graft viability in the Ecosol groups was better preserved as illustrated by the IVIS imaging results, despite lacking statistical significance. This is most likely due to the high standard deviations especially among UW groups as well as the relatively low total number of transplanted grafts among all groups. Apoptosis was less likely to occur when Ecosol was used, as displayed by the TUNEL Assay scores, despite lacking statistical significance.

### 3.1. Preservation of the Bile Duct

Biliary complications account for the vast majority of postoperative complications in liver transplantation. Bile leakage and biloma along with cholangitis appear to be the most frequent complications [23]. Our findings showed significantly lower total bilirubin values on day 7 post-transplantation when Ecosol was used compared to UW among syngeneic groups. Biloma more frequently occurred in both UW groups compared to Ecosol. One possible explanation can be the high viscosity of the UW solution, as high viscosity hinders the perfusion of the bile canaliculi and therefore the bile duct is not fully protected and preserved during cold storage. Therefore, a low viscosity solution may be more beneficial. GGT was significantly lower for Ecosol compared to UW in syngeneic settings on day 1 post-transplantation. These results are in line with previous reports, where lower viscosity solutions were able to better preserve the biliary tract [24].

### 3.2. Baseline Values

When comparing AST and ALT to baseline values in the allogeneic transplant group, Ecosol treated livers recovered faster when compared to UW. On day 7 post-transplantation, AST and ALT values for Ecosol did not show any statistically significant differences to the baseline values of BN rats, while values for UW treated livers remained significantly higher on day 7 of transplantation.

In the syngeneic group, similar results were achieved. AST values on day 7 post-transplantation remained significantly higher for UW compared to the known baseline values of Lewis rats, while Ecosol did not show any difference. Our findings in allogenic and syngeneic groups imply that immunocompatibility is a secondary point of concern, especially in liver transplantation, when aiming to improve graft quality. As AST and ALT are both clinically applied markers for hepatocyte injury, we can assume that Ecosol performed better in hepatocyte protection and recovery after the negative impacts of the cold ischemic injury to the liver grafts compared to UW.

### 3.3. Graft Viability

The quantification of transgenic luciferase rats has been widely applied for the evaluation of graft viability in liver preservation during cold storage and has been proven to be highly effective [25]. Briefly, it was shown that luminescence intensity measured by an in vivo imaging system directly reflects intracellular ATP levels and thus hepatocyte viability. We used the IVIS imaging system for the detection of bioluminescence emission after 8 h of cold storage in the respective solution. Luciferase expression was stronger for Ecosol compared to UW, which indicates a better preservation of liver grafts during SCS. We considered the limitations for the IVIS imaging system, e.g., signal interference.

### 3.4. Apoptotic Changes

We already emphasized the importance of ischemia–reperfusion injury to liver grafts. IRI seems to be dominated by apoptosis, in addition to necrotic processes similarly occurring [26]. Such apoptotic changes on the molecular level were meant to be quantified and analyzed by means of the TUNEL Assay. Our results imply that apoptosis is more likely to occur within UW solution treated liver grafts compared to Ecosol in both syngeneic and allogenic groups. Apoptotic processes seem to be less likely when antioxidants are major components of the preservation solution during SCS [27]. Ecosol, being such a solution, contains taurine, one of the strongest known antioxidants, instead of allopurinol (UW), whereas glutathione is present in both (gluthatione concentration is 12–30 times higher in Ecosol compared to UW). Both taurine and gluthatione act as strong scavengers for reactive oxygen species (ROS), especially during IRI, and thus reduce the mitochondrial damage in hepatocytes. This mechanism reduces the negative impacts of IRI on hepatocytes and enables the faster normalization of AST/ALT after transplantation. This mechanism was previously shown in our working group during the normothermic perfusion of porcine kidneys, showing an increased antioxidant capacity in the Ecosol group, accompanied by a decreased oxidation reduction potential [28]. PEG is another major component of Ecosol, which reduces oxidative stress and inflammatory processes as well as supporting mitochondrial stability during static cold storage. Functionally, PEG facilitates the conservation of hepatic ATP reserves and sustains a favorable ATP/ADP balance. Upon reperfusion, the activation of the adenosine-monospohpate kinase (AMPK) is promoted, while lactate accumulation in the perfusate is diminished. This constellation of effects suggests a reduced reliance on anaerobic glycolysis and an enhanced mitochondrial oxidative metabolism compared to conventional preservation solutions lacking PEG [29].

### 3.5. Study Limitations

Our study is limited by the short follow-up (7 days as endpoint) and thus data on, e.g., biliary strictures or fibrotic changes are not available. Expanded endpoints shall be considered in future studies. We prioritized the measurement of serum AST, ALT, LDH, ALP, and GGT due to the limited amount of blood serum in this rat study, compared to the ex vivo perfusion of porcine kidneys. Further laboratory values of interest and immunological markers may be obtained and evaluated in the future. Additionally, larger sample sizes, mechanistic studies (e.g., ROS quantification), and porcine DCD models may be considered for clinical relevance.

## 4. Materials and Methods

### 4.1. Experimental Design

The study was performed in accordance with the German animal welfare law (Tierschutzgesetz (TSchG)) and the EU-Directive 2010/63/EU [30]. The Governmental Animal Care and Use Committee approved the experiment protocol (Reference No.: 84-02.04.2014.A487; Landesamt für Natur, Umwelt und Vebraucherschutz Recklinghausen, North Rhine Westphalia, Germany). According to FELASA guidelines, all employees were trained and instructed on care and rat-specific behavior. We used male Lewis or Brown Norway rats (Janvier S.A.S., Saint-Berthevin Cedex, France), with an age of 6–8 weeks (200–250 g body weight), according to a priori power calculation for group size. Additionally, genetically modified luciferase rats (Luc tg- Lewis rats) were used for in vivo imaging of the liver before and after perfusion. The animals were randomly assigned (www.randomizer.org) and group-housed in filter-top cages (Type 2000, Tecniplast, Buguggiate, Italy) in a controlled environment (12 h/12 h light–dark cycle; temperature: 22 °C ± 2 °C; relative humidity: 50% ± 20%) under SPF conditions according to the FELASA guidelines [31]. The experiments took place time-shifted, so that housed and operated animals were not in the same room simultaneously to avoid interference by pheromones. Red play tunnels (tunnel Ø 155 × 75 mm, #3084014, Zoonlab GmbH, Castrop-Rauxel, Germany) for handling and cage enrichment as well as low-dust wood granulate as bedding (Rettenmeier Holding AG, Wilburgstetten, Germany) as well as nestlets as enrichment were used. We followed the ARRIVE guidelines for reasons of reliability of our study [32].

**Figure 5:**
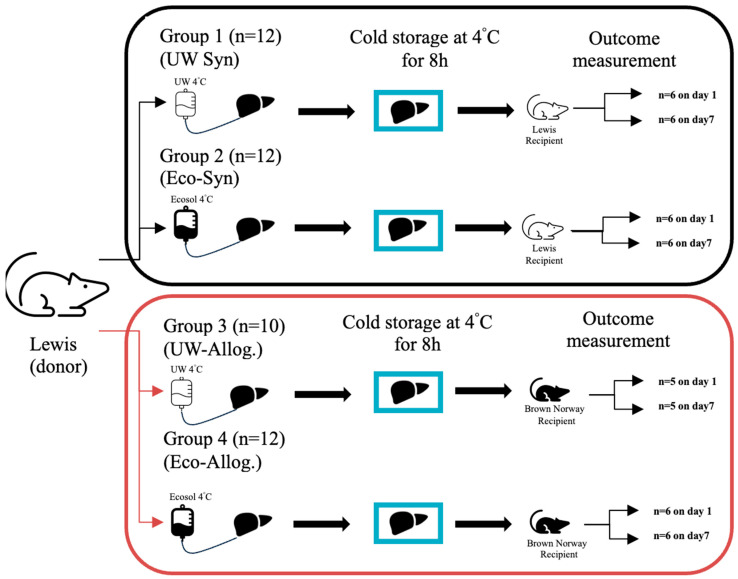
Experimental design.

We conducted the study by forming an allogenic and syngeneic group. Male Lewis rats were the sole liver donors. In the allogenic group, Brown Norway rats were the recipients. Donor as well as recipient rats were 6–8 weeks old; the animals had ad libitum access to sterile food (rat diet, Sniff, Soest, Germany) and water. Within each group, before transplantation, donor livers were preserved either with the novel Ecosol preservation solution or UW solution (control) and cold stored at 4 °C for 8 h. Two analysis time points were set: 24 h (1 day) and 168 h (7 days) after transplantation (Figure 5).

### 4.2. Experimental Procedure and Surgical Approach

General anesthesia was induced by using Isoflurane/buprenorphine (1.5 Vol% Isoflurane/buprenorphine 0.1 mg/kg, s.c., Forane; Abbott GmbH, Wiesbaden, Germany/Temgesic; EssexPharma, Haar, Germany) 30 min prior to surgery. Donor and recipient operation was described by our group previously [33,34,35,36,37]. Briefly, after a median laparotomy with subcostal extension, the livers were explanted. Following the preparation and cannulation of the blood vessels, donor livers were perfused via the portal vein in vivo with 15 mL of the 4 °C cold preservation solution, either Ecosol or UW. The explanted livers were stored within their respective solutions for 8 h. Hereafter, livers were transplanted to the recipient, either Lewis rats in the syngeneic or Brown Norway rats in the allogenic transplant group. All recipients received an antibiotic prophylaxis with Cefuroxime (16 mg/kg BW, s.c. Cefuroxim Fresenius; Fresenius Kabi Deutschland GmbH, Bad Homburg, Germany) during transplantation. In the allogenic groups, Brown Norway rats additionally received an immunosuppressive therapy using Cyclosporin A (2.0 mg/kg BW, s.c., Sandimmun 100 mg/mL, Novartis Pharma GmbH, Nürnberg, Germany) and Hydrocortisone (0.75 mg/kg BW, s.c., 100 mg Hydrocortisone powder, Pfizer Pharma GmbH, Berlin, Germany) every 24 h throughout the time of observation. Analgesia was provided with Buprenorphine, 0.1 mg/kg BW s.c., twice daily for three postoperative days. Upon successful transplantation, recipient rats were evaluated twice daily in both groups. This was in line with postoperative clinical scoring, evaluating severity and humane endpoints. At the end of the observation period (24 h or 168 h), recipient rats were placed under general anesthesia as described above, and a relaparotomy was performed. Laboratory diagnostics of liver enzymes and histology (#MBS2021174, My Biosource, Inc., San Diego, CA, USA) were conducted. Rats were then euthanized by exsanguination as well as organ removal for analysis under deep general anesthesia.

### 4.3. Obtained Parameters

Comprehensive serum analysis was performed, with a focus on AST (U/L), ALT (U/L), LDH (U/L), ALP (U/L), GGT (U/L), and total bilirubin (µmol/L) (Clinical Chemistry Analyzer, Vitros 250; Johnson and Johnson, Neuss, Germany). Samples were taken and measured after 24 h and 168 h of transplantation in the respective groups. At the same timepoints, serum values were compared to baseline values of Lewis (Data sheet of LEW/OrlRj, Janvier Labs, Le Genest Saint Isle, France) and Brown Norway rats (historical data of approved protocol #81-02.04.2018.A155). 

We assessed apoptotic lesions on histological samples via TUNEL Assay (TUNEL, terminal deoxynucleotidyl transferase biotin-dUTP nick end labeling Assay Kit—HRP-DAB, #ab206386, Abcam, Cambridge, UK) 24 h after transplantation.

We utilized livers from Luciferase expressing Lewis transgenic rats to analyze the organ quality after preservation by using the photonic IVIS imaging system (In Vivo Imaging, Revvity Germany Diagnostics, Lübeck, Germany). Livers were explanted as described above and perfused with either Ecosol or UW solution. Live imaging was performed initial after explantation and at 8 h after cold storage time in the respective organ preservation solution for comparison.

### 4.4. Statistical Analysis

Statistical analysis was performed using GraphPad Prism 10 (GraphPad Software Inc., San Diego, CA, USA). The primary statistical analysis was performed using one-way or two-way ANOVA (factors: group and time) on the raw data per animal. The assumption of normality was assessed using Q-Q plots of the model residuals and visual diagnostics. Statistical significance was considered at *p* < 0.05. Data were given as mean ± SD unless otherwise stated.

## 5. Conclusions

Ecosol proved biological safety and non-inferiority according to the Medical Device regulation compared to the gold standard UW for liver preservation in a rat orthotopic liver transplantation model. In the allogenic settings, Ecosol treated livers recovered faster and better, when compared to the baseline values. Liver grafts were equally well or better preserved with Ecosol, as shown by liver function tests. Recovery after transplantation was faster in Ecosol groups compared to UW in both syngeneic and allogenic settings. Apoptosis occurred less in the Ecosol treated livers compared to the UW groups. Further studies in large animals with larger study populations are warranted.

## Figures and Tables

**Table 1:** Comparison of Ecosol solution with the UW solution [16,17,18].

Component	Ingredients	Ecosol	UW
Colloid Impermeants Buffers	PEG 35,000	5.4	-
	HAES 5%	-	50 g/L
	Sodium gluconate	37.1	-
	Magnesium gluconate/sulfate	12.0	1.23 g/L
	Calcium gluconate	2.3	-
	Lactobionic acid	12.0	35.8 g/L
	Trehalose	4.0	-
	Raffinose	1.7	17.8 g/L
	HEPES	16.8	-
	Histidine	10.0	-
	Potassium phosphate	2.2	3.4 g/L
	Sodium bicarbonate	2.2	-
	Sodium citrate	3.1	-
	Potassium hydroxide	-	5.6 g/L
Antioxidants Energy substrates	Taurine	36.8	-
	Glutathion	12.0	0.9 g/L
	Allopurinol	-	0.1 g/L
	Glucose	6.9	-
	Pyruvate	1.8	-
	Adenosine	6.0	1.3 g/L
Amino acids	Tryptophan, Arginine, Carnitine, Cysteine, Glutamic acid, Glutamine, Glycine, Ornithine	Yes	No
Vitamins	Ascorbic acid, Biotin	Yes	No
Electrolytes	Na+/K+	124/12	29/125
Viscosity	at approximately room temperature	2.2 c.P.	5.7 c.P.
Osmolarity		310 mOsmol/L	320 mOsmol/L

All values in mmol/L unless indicated otherwise in the respective column. Different manufacturers may have small deviations in concentration of components. Belzer UW^®^ Cold Storage Solution from Bridge to Life (Europe Ltd., Wandsworth, UK).

## Data Availability

The original contributions presented in this study are included in the article. Further inquiries can be directed to the corresponding author.
